# Long term crop rotation effect on subsequent soybean yield explained by soil and root-associated microbiomes and soil health indicators

**DOI:** 10.1038/s41598-021-88784-6

**Published:** 2021-04-28

**Authors:** Achal Neupane, Izzet Bulbul, Ziyi Wang, R. Michael Lehman, Emerson Nafziger, Shin-Yi Lee Marzano

**Affiliations:** 1 Department of Biology and Microbiology, South Dakota State University, Brookings, SD 57007 USA; 2 Department of Agronomy, Horticulture, and Plant Science, South Dakota State University, Brookings, SD 57007 USA; 3 United States Department of Agriculture, Agricultural Research Service, North Central Agricultural Research Laboratory, Brookings, SD 57006 USA; 4 Department of Crop Sciences, University of Illinois, Urbana, IL 61801 USA; 5 United States Department of Agriculture, Agricultural Research Service, Application Technology Research Unit, Toledo, OH 43606 USA

**Keywords:** Agroecology, Biogeochemistry, Microbial ecology

## Abstract

Crop rotation is an important management tactic that farmers use to manage crop production and reduce pests and diseases. Long-term crop rotations may select groups of microbes that form beneficial or pathogenic associations with the following crops, which could explain observed crop yield differences with different crop sequences. To test this hypothesis, we used two locations each with four long-term (12–14-year), replicated, rotation treatments: continuous corn (CCC), corn/corn/soybean (SCC), corn/soybean (CSC), and soybean/corn (SCS). Afterwards, soybean was planted, and yield and soil health indicators, bulk soil microbiome, and soybean root-associated microbiome were assessed. Soybean yields, as well as soil protein, and POXC as soil health indicators were higher following CCC than in the other three treatments at both locations. A bacterial taxon in family *JG30-KF-AS9* was enriched in CCC, whereas *Microvirga*, *Rhodomicrobium*, and *Micromonosporaceae* were enriched in SCS. Several ascomycetes explain lowered yield as soybean pathogens in SCS. Surprisingly, *Tumularia*, *Pyrenochaetopsis* and *Schizothecium* were enriched in soybean roots after CCC, suggesting corn pathogens colonizing soybean roots as nonpathogens. Our finding of associations between soil health indicators related to microbiomes and soybean yield has wide-ranging implications, opening the possibility of manipulating microbiomes to improve crop yield potential.

## Introduction

Both the composition and function of microbial communities can be substantially manipulated by management tactics for “smart farming”^1^. Cumulative management effects can be identified by long-term experiments which help to identify problems that threaten future productivity as an early warning system^2^, explain the reasons behind existing agricultural production problems^3^, and assist in formulating solutions. Additionally, it is important to understand the cumulative effects of enduring management strategies in order to sustain optimum soil properties^4^, specifically the effects on microbial communities and soil health as a result of crop rotation sequences.

Shifts in plant or soil-associated microbial communities are driven by a myriad array of legacy and emerging factors such as plant genetics, soil chemical/physical properties and environmental conditions or soil processes^5^. Because the microbiome is an integral part of almost all soil processes^6^, the structure of the microbial communities associated with soil and plants can be directly affected by management strategies such as crop rotation sequences^7^. In the Midwest U.S., corn (*Zea mays*) and soybean (*Glycine max*) cover about 75% of acres used to grow crops^8^ and are commonly grown in rotation, which generally improves yields of both crops. However, shifts in the resulting microbial communities due to this crop rotation and variations in crop sequencing are unclear, and may explain yield differences as well as provide new knowledge for future yield improvements.

We selected three of the most biologically-oriented, dynamic soil health indicators that have been recently recommended for routine use in agricultural soils by United States Department of Agriculture Natural Resources Conservation Service^9^. These three measurements described below, represent the top recommendations for indicators of organic C availability, organic N availability, and general organic decomposition activities^9^. The amount of permanganate-oxidizable carbon (POXC) represents the labile portion of the organic C which is the most reactive and dynamic driver of C mineralization within the pool of soil organic C (SOC)^10,11^. Labile organic C as measured by POXC has been directly associated with soil C and N mineralization^12^, and may promote plant productivity due to its positive influence on soil activities and nutrient availability^13^. Positive correlations have been found between POXC and soil-microbial parameters, comprising microbial biomass and, in particular organic C^10,14^. Therefore, POXC is the recommended method for C food source of microbes. A second soil health indicator, the autoclaved citrate extractable (ACE) protein content, refers to bioavailable N in the soil organic matter (SOM)^15^. The largest organic N pool in the soil is represented by proteins^16–18^. The labile organic N pool is used to evaluate soils’ capacity to provide N^19^ by promoting N mineralization in soils^20^. Regarding plant growth and development, N mineralization is a critical process in the soil to provide an adequate amount of N for the use of the plant^21^. Since protein content is an indicator of biological and chemical soil health, especially for SOM quality, it is directly linked to general soil health status^22^. Soil protein includes an N-linked glycoprotein, glomalin, which is produced by arbuscular mycorrhizal fungi hyphae^23,24^. Glomalin has been reported to enhance soil structure, drainage, microbial activity, and C sequestration in soil ecosystems^25^ and is sensitive to crop rotation and tillage^22,26–28^. A third soil health indicator, β-glucosidase, is an enzyme which plays a central role in the C cycle in soil^29^ and serves as an important indicator of general microbial activity^30^. In terms of the C cycle, the importance of soil microorganisms in many ecosystems hinges on breaking down cellulose in plant cell walls^31^; cellulose is one of most common organic compounds in the biosphere^32^. β-glucosidase, which has a role in the final stage of cellulose degradation in soils, supplies important energy sources, like simple sugar, for microorganisms^33^. A variety of microorganisms are involved in β-glucosidase activity in soils including filamentous fungi^34–42^, yeast^43^, and bacteria^44,45^.

The lack of strong correlations between rotation-induced crop yield differences and soil chemical and physical properties suggest that soil-associated and plant-associated microbiomes could be determinants for these differences^4,46^. Since soil bacteria and fungi directly mediate the C and N cycles, and regulate the nutrient availability for plants, soil health indicators are expected to be correlated with members of the soil microbiome. We hypothesized that crop yield differences that result from crop rotation would correspond to soil health indicators and soil or root-associated microbiome. To test this hypothesis, we used two sites, each with three replicated, long-term (12–14 year) crop rotations: continuous corn (CCC), corn/corn/soybean (SCC), and corn/soybean (CSC), with each phase of CCS and CSC present each year. In total, there were 4 CCS cycles, 6 SC cycles, and 12 CCC cycles, but corn was in place 2 years prior to the other rotation schemes were fully established. Soil samples were taken right before the soybean was planted the sampling year. In Year 15 (the sampling year) when the continuous corn had been in place for 14 years, soybean was planted in all plots, producing treatments with 14 (CCC), 2 (SCC), and 1 (CSC) year of corn, and of one year (SCS) of soybean as the previous crop. Samples were collected for analyses of soil and root-associated microbiome, soil health indicators, and soybean yield.

## Results

### Soybean yield and soil health indicators

Data for soybean yield, soil protein, POXC, and β-glucosidase are summarized in Table 1. At both sites there were significant differences in soybean yield following the long-term crop sequences of CCC, SCC, CSC, and SCS. Soybean yields from CCC (5614 and 5256 kg ha^−1^ at Urbana and Monmouth, respectively) were significantly higher than SCC, CSC, and SCS at both sites. At Urbana, SCC (with two previous years of corn) yielded more than SCS, with the previous crop of soybean. At the Monmouth site, there were no significant differences among the SCC, CSC, and SCS treatments, but yields trended lower as the proportion of corn crops prior to the soybean crop decreased, and were lowest where soybean was the previous crop. Across both sites, the rotations with a higher frequency of corn (CCC, SCC) produced higher yields of soybean than those with corn in alternate years (CSC, SCS).

**Table 1:** Means of soybean yield, soil protein, permanganate-oxidizable carbon and β-glucosidase affected by crop rotation.

Treatment	Yield (kg/ha^−1^)		Protein (mg/kg soil)		POXC (mg C/kg soil)		β-glucosidase (mg p-nitrophenol/gm)	
	Urbana	Monmouth	Urbana	Monmouth	Urbana	Monmouth	Urbana	Monmouth
CCC	5614 aA	5256 aA	7360 aA	5842 aA	647 abA	816 aA	0.93 bB	1.51 aA
SCC	5010 bA	4802 bA	5627 bA	5426 aA	777 aA	734 aA	0.91 bB	1.24 bA
CSC	4803 cbA	4650 bA	5417 bA	5453 aA	490 bA	606 bA	0.87 bB	1.73 aA
SCS	4719 cA	4598 bA	5613 bA	5469 aA	562 bA	585 bA	1.19 aB	1.73 aA

Crop rotation: CCC (continuous corn), SCC (2 years corn), CSC (1 year corn) and SCS (1 year soybean). Means within the same column followed by different lower-case letters are significantly different at *P* < 0.05 for rotation. Means within the same row followed by different capital letters are significantly different at *P* < 0.05 for the two locations.

Soils in the CCC treatment had higher protein content compared with other crop sequences at Urbana. The CCC (7360 mg per kg soil) treatment was significantly greater than SCC (5627 mg per kg soil), CSC (5417 mg per kg soil) and SCS (5613 mg per kg soil) treatments (*p* < 0.05). There were no significant differences among the SCC, CSC and SCS treatments. At the Monmouth location, we found no significant differences in protein content among the CCC (5,842 mg per kg soil), SCC (5426 mg per kg soil), CSC (5453 mg per kg soil) and SCS (5,469 mg per kg soil) crop sequences, although CCC had the highest (numerically) average protein.

Soil β-glucosidase enzyme activity was significantly higher in SCS (Urbana: 1.19 and Monmouth: 1.73 mg p-nitrophenol per g of soil) treatments compared with other rotations (*p* < 0.05) at both sites. We found no differences among the CCC (0.93 mg p-nitrophenol per g soil), SCC (0.91 mg p-nitrophenol per g of soil) and CSC (0.87 mg p-nitrophenol per g of dried soil) rotations at the Urbana site. At Monmouth, SCS (1.73 mg p-nitrophenol per g of soil) and CSC (1.73 mg p-nitrophenol per g of soil) had more β-glucosidase enzyme activity than CCC (1.51 mg p-nitrophenol per g of soil) and SCC (1.24 mg p-nitrophenol per g of soil). Distribution analysis showed that soil β-glucosidase activity values were normally distributed.

After 12–14 years of the above-mentioned crop rotation regimes, we found that at Monmouth site POXC was significantly greater in the CCC (816 mg C per kg soil) and SCC (734 mg C per kg soil) (*P* < 0.001) treatments than in the CSC and SCS treatments (Table 1). There was no difference in POXC between CSC (606 mg C per kg soil) and SCS (585 mg C per kg soil) treatments. At the Urbana site, POXC was significantly higher in CCC (647 mg C per kg soil) and SCC (777 mg C per kg soil) compared to the SCS (562 mg C per kg soil) and CSC (490 mg C per kg soil) treatments (*P* < 0.05). Differences between the CCC and SCC, and among the CCC, CSC, and SCS treatments were not significant.

Analysis across sites with site treated as a random effect, there were no significant differences in soybean yield, soil protein and POXC between the Urbana and Monmouth locations for the same treatments. However, β-glucosidase enzyme analysis was significantly different for the same treatments at the two locations.

### Bulk soil microbiome

**Figure 1:**
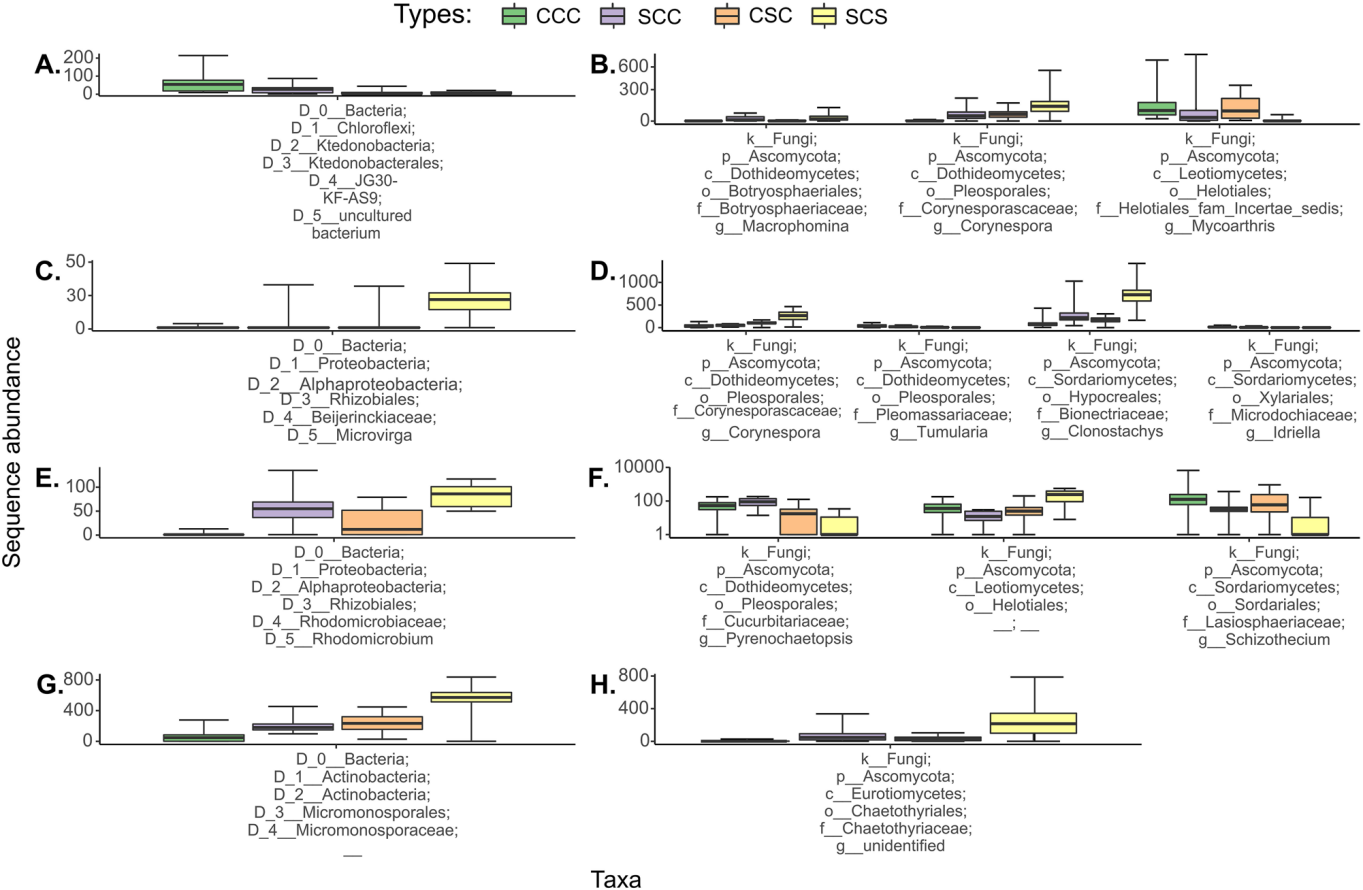
Differential abundance of taxa analyzed by ANCOM approach. Box-and-whisker plots of bacterial (**A**) and fungal (**B**) abundance at the Monmouth site, and bacterial (**C**) and fungal (**D**) abundance at the Urbana site associated with bulk soil; bacterial (**E**) and fungal (**F**) abundance at the Monmouth site, and bacterial (**G**) and fungal (**H**) abundance at the Urbana site associated with roots are shown with relative abundance distributions for the taxa that varied significantly among rotation treatments.

Based on ANCOM results in the bulk soil data from Monmouth, an uncultured bacterium belonging to the order of JG30-KF-AS9 within the *Chloroflexi* phylum had a descending relative abundance order of CCC > SCC > CSC > SCS (Fig. 1A). In the fungal community in the bulk soil from the same site, the relative abundance of *Ascomycota* effectively discriminated among the four crop sequences. Specifically, the *Macrophomina* genus was detected as the most abundant in the SCS crop sequence with a decreasing order of abundance as SCS > SCC > CSC > CCC (Fig. 1B). The relative abundance of the genus of *Corynespora* was significantly higher in the SCS rotation with a decreasing relative abundance in the order of SCS > SCC > CSC > CCC rotations. The genus *Mycoarthris* was more abundant in the SCC and CCC rotation groups; and less abundant in the two-year rotation treatments CSC and SCS.

In the bulk soil from the Urbana site, the bacterial genus *Microvirga*, belonging to family *Beijerincklaceae* under class *Alphaproteobacteria*, was an informative taxon distinguishing the four treatments, with higher abundance in low-yield rotation groups (CSC and SCS) compared to high-yield (CCC and SCC) rotation groups (Fig. 1C). An uncultured fungus belonging to family *Corynesporascaceae* in order *Pleosporales* under class *Dothideomycetes* in the phylum of *Ascomycota* was found significantly different in relative abundance separating the four crop sequences, with the order of SCS > CSC > CCC > SCC at the Urbana site (Fig. 1D). From the same order, a taxon under family *Pleomassariaceae* and genus *Tumularia* has been found significantly different in relative abundance in decreasing order of CCC > SCC > CSC > SCS. Two additional taxa from genus *Clonostachys* and *Idriella* under class *Sordariomycetes* and phylum *Ascomycota* were found to be significantly different in terms of relative abundance. *Clonostachys* was found to be in a decreasing order of SCS > SCC > CCC > CSC, while *Idriella* was higher in abundance in high-yield groups in the decreasing order of CCC > SCC > CSC > SCS.

### Root-associated microbiome

The most informative root-associated bacterial genus at the Monmouth site was *Rhodomicrobium* under class *Alphaproteobacteria* which has significantly different in relative abundance among the four crop sequences in the order of SCC > SCS > CSC > CCC (Fig. 1E). For fungal community from the same site, three taxa all belonging to phylum *Ascomyota* under order *Pleosporales* (genus *Pyrenochaetopsis*), *Leotiomycetes* (genus unknown) and *Sordariales* (genus *Schizothecium*) were found to be significantly different in relative abundance with the order of CCC > SCC > CSC > SCS, SCS > CSC > CCC > SCC and CCC > CSC > SCC > SCS, respectively (Fig. 1F). At the Urbana site, a root associated bacterium that was differentially abundant among the rotations was *Micromonosporaceae* under phylum *Actinobacteria* with the order of increasing previous crop corn frequency: SCS > CSC > SCC > CCC (Fig. 1G). A root-associated uncultured fungus belonging to family *Chaetothyriaceae* in order *Chaetothyriales* under class *Eurotiomycetes* in the phylum of *Ascomycota* was found to be the only differential fungal taxa at the Urbana site and had the greatest abundance in the SCS crop sequence and a decreasing abundance order of SCS > SCC > CSC > CCC (Fig. 1H).

**Figure 2:**
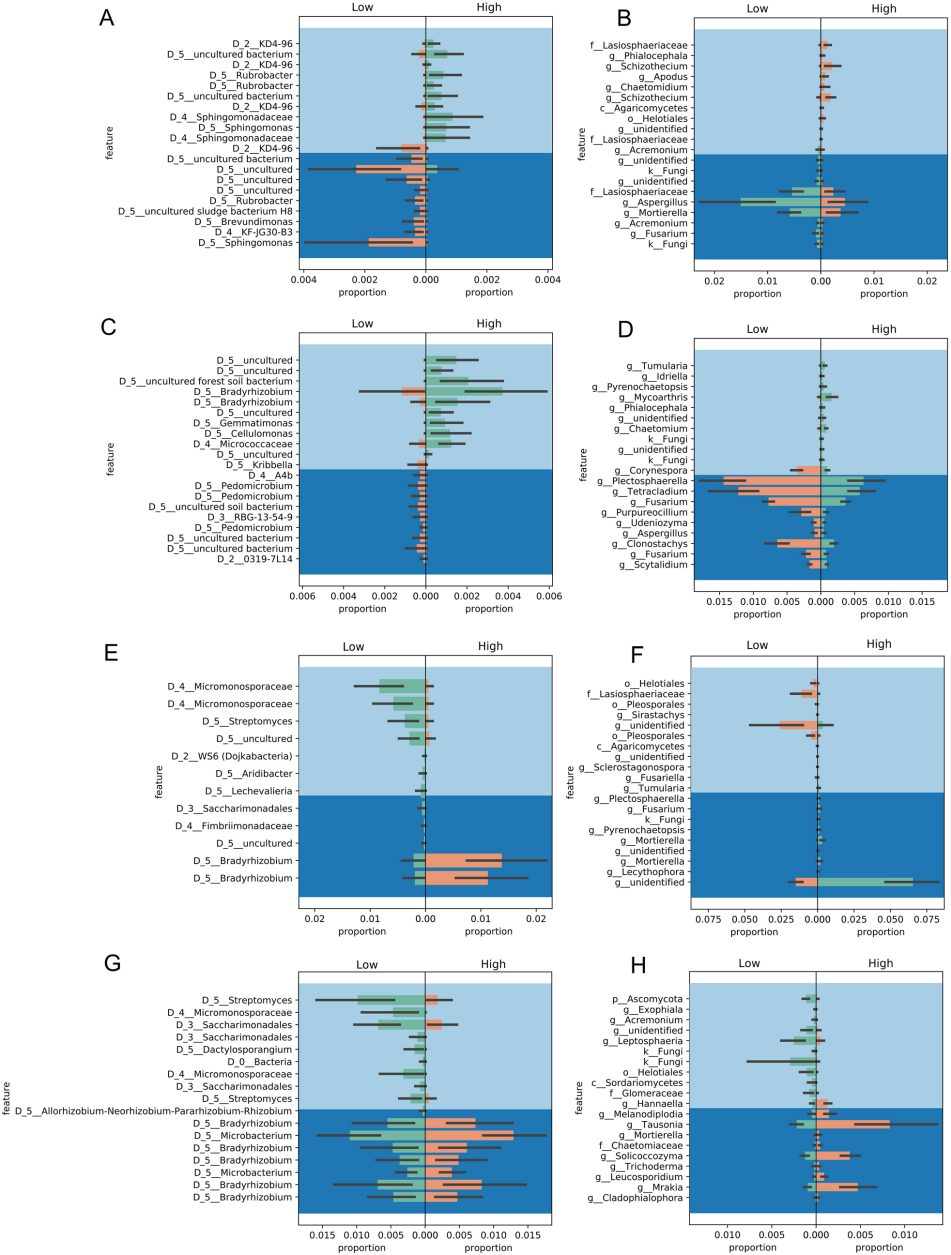
Differential abundance of taxa analyzed by Gneiss approach. Abundances of bacterial (**A**) and fungal (**B**) population at the Monmouth site, and bacterial (**C**) and fungal (**D**) population at the Urbana site associated with bulk soil; bacterial (**E**) and fungal (**F**) population at the Monmouth site, and bacterial (**G**) and fungal (**H**) population at the Urbana site associated with roots are shown. The differential abundance analysis was performed based on high- and low-yield groups. The high yield group is CCC (continuous corn) and SCC (two years corn). The low yield group is CSC (one year corn) and SCS (one year soybean). Figure was created using built-in function in^85^.

Following the ANCOM analysis, differential abundance analysis of taxa between higher-yielding (CCC and SCC) and lower-yielding (CSC and SCS) soybean crops was performed using balances in Gneiss. In the bulk soil from Monmouth, members of the genus *Rubrobacter* were proportionally higher in the high-yield treatments and *Sphingomonas* were proportionally higher in the low-yield treatments (Fig. 2A). Fungi from the genus *Aspergillus* were proportionally higher in the low-yield rotation groups (Fig. 2B). At Urbana, bacterial genera *Bradyrhizobium*, *Gemmatimonas*, *Cellulomonas*, family *Micrococcaceae*, and several uncultured taxa were proportionally higher in high-yield rotation groups (Fig. 2C), while several fungi from genera *Plectosphaerella*, *Tetracladium*, *Fusarium*, *Clonostachys*, and *Purpureocillium* were present in higher proportions in low-yield rotation groups (Fig. 2D).

Root-associated microbiome communities from Monmouth, however, showed that *Actinobacteria* from the family *Micromonosporacea* and genus *Streptomyces* were proportionally higher in low-yield rotation groups and bacteria from genus *Bradyrhizobium* were proportionally higher in high-yield rotation groups (Fig. 2E). Fungi from the family *Lasiosphaeriaceae* were found in higher proportion in low-yielding rotations, including some unidentified fungi in both high- and low-yield rotation groups (Fig. 2F). At Urbana, several bacteria belonging to genus *Streptomyces*, family *Micromonosporaceae*, which are both under phylum *Actinobacteria*, and order *Sacharimonadales* were in higher proportions in low-yield treatments (Fig. 2G). Root-associated fungi from Urbana, such as genera *Tausonia*, *Solicoccozyma*, and *Cladophialophora* in high-yield groups were found in higher proportion, whereas *Leptosphaeria* and several uncultured fungal taxa were more abundant in the low-yield groups (Fig. 2H).

Rarefaction curves were graphed to visualize the minimum amount of sequencing reads required for the analysis. In Figure S1, the X-axes represent the number of sequences extracted from each sample and the Y-axes represent the alpha diversity based on the Shannon index. The rarefaction curves for the four rotation groups plateaued for both soil (Figure S1; A-D) and root-associated (Figure S1; E–H) microbial communities, indicating that the sequencing depth (as a function of sampling) was sufficient and any incremental change in the reads of sequencing data would not have further contributed to the species diversity or discovery of any additional species.

**Figure 3:**
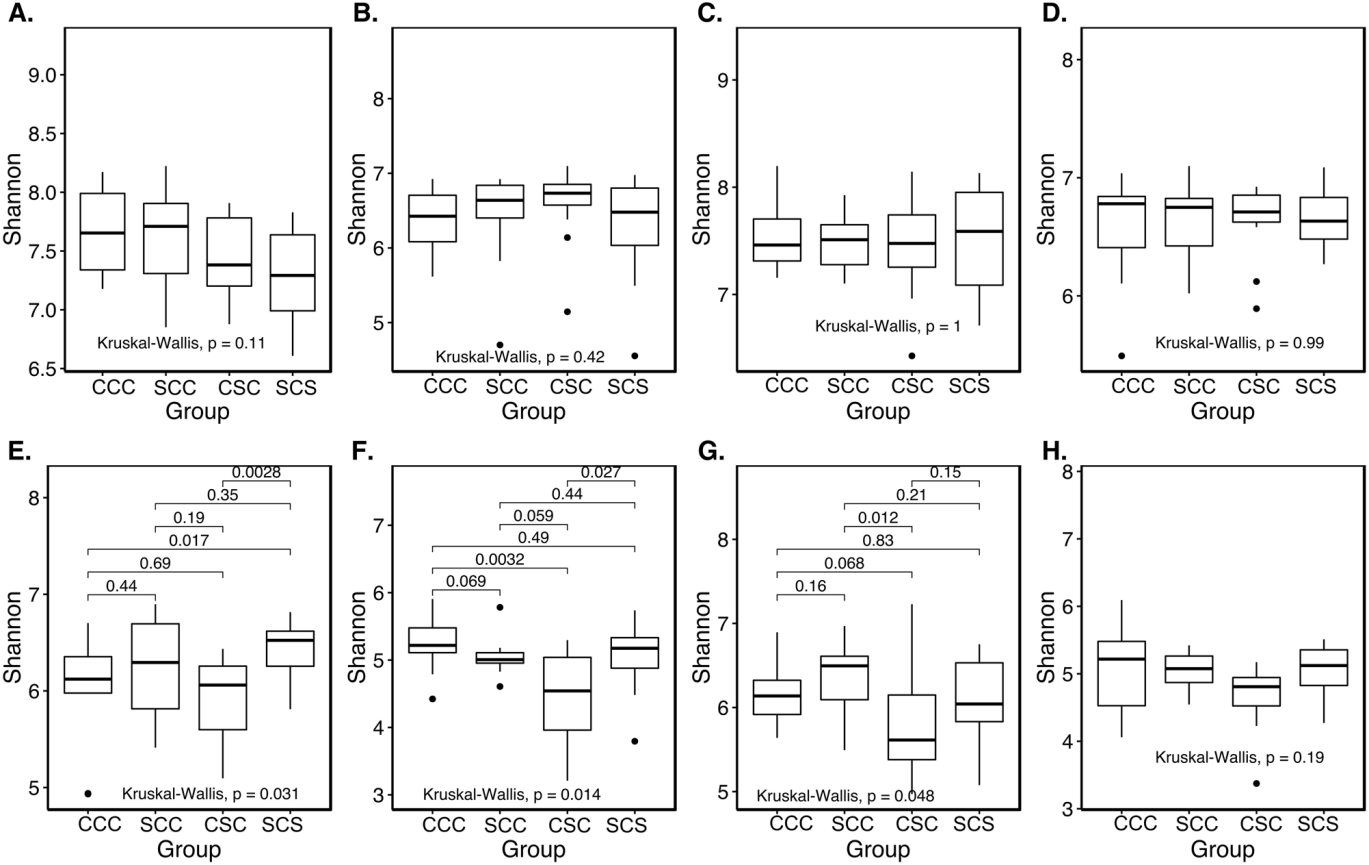
Alpha diversity estimated by the Shannon index. Bacterial (**A**) and fungal (**B**) diversities at the Monmouth site, and bacterial (**C**) and fungal (**D**) diversities at the Urbana site associated with bulk soil; bacterial (**E**) and fungal (**F**) diversities at the Monmouth site, and bacterial (**G**) and fungal (H) diversities at the Urbana site associated with roots are shown.

With data combined between the two locations for the analysis of alpha diversity, there were no significant differences among the treatments analyzed with either bulk soil or root-associated microbiome data. Data was also analyzed separately for the two locations, and no differences were found in the bulk soil microbiome based on the Shannon index (*P* > 0.05) among the four rotations (Fig. 3A–D). However, significant differences (*P* < 0.05) were found among the four treatments in the Shannon index for root-associated bacterial (Fig. 3E) and fungal (Fig. 3F) communities from Monmouth. Also, root-associated bacterial communities were found to be significantly different among treatments at Urbana (Fig. 3G), whereas fungal communities were not significantly different among the four treatments (Figure S2; 3H). Interestingly, CSC rotation had consistently the lowest average diversity in the root-associated microbiome.

Beta-diversity analyses of the bacterial communities from bulk soil did not produce separate clusters for the four rotation treatments (Figure S2; A and C) but did for the fungal communities (Figure S2; B and D). On the other hand, beta-diversity of both bacterial and fungal communities associated with roots did produce separate clusters for four rotation treatments (Figure S2; E–H).

**Figure 4:**
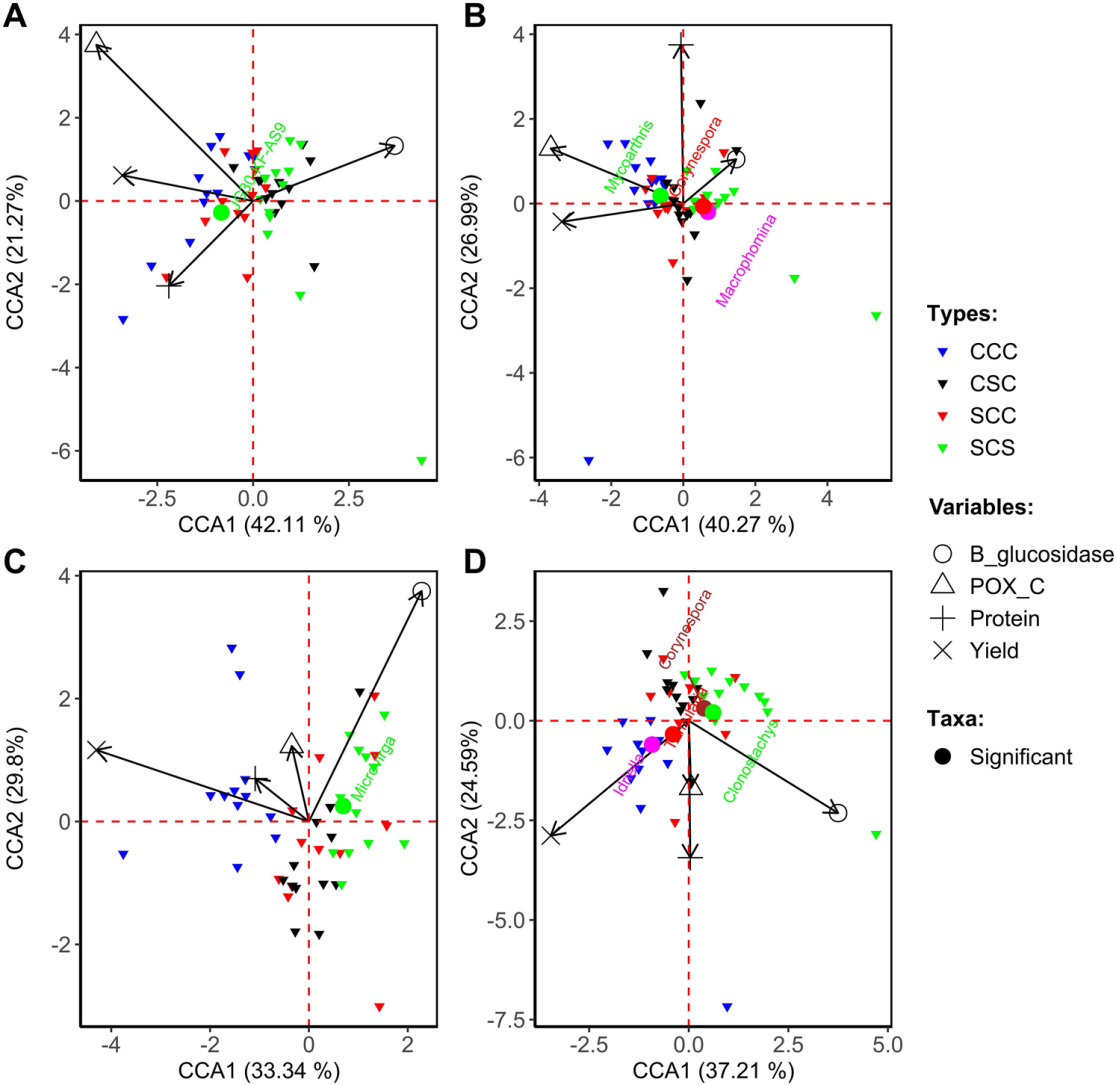
Relationships between operational taxonomic units from different crop rotations and the soil health indicators and soybean yield. A multivariate approach using canonical correspondence analysis (CCA) was used. Only significant taxa are shown by colored-coded circles. Rotation regimes are denoted by color-coded triangles (Treatments) and soil health indicators are denoted by hollow shapes (Indicators). Bacterial (**A**) and fungal (**B**) taxa with differential abundances at the Monmouth location, and CCA for bacterial (**C**) and fungal (**D**) taxa with differential abundances at the Urbana location are shown.

To correlate the soil health indicators with bulk soil microbiomes and the crop rotation treatments, the results of canonical correspondence analysis (CCA) were plotted. The rotations that preceded higher-yielding soybean (CCC and SCC) were associated with POX-C and protein, while those preceding lower-yielding regimes (CSC and SCS) were associated with β-glucosidase (Fig. 4A–D). We did not see strong association of significantly abundant microbial taxa with any of the soil health indicators analyzed for Monmouth and Urbana sites (Fig. 4A–D).

## Discussion

Studies have shown higher soybean yields when soybean follows grain crops such as corn or sorghum instead of soybean, with two or more years of corn as the previous crop sequence often producing higher soybean yields than a single corn crop^47–50^. Similarly, we found that 12–14 years of continuous corn produced higher soybean yields than fewer years, and two years producing marginally higher soybean yields than one year of corn preceding soybeans. A recent study conducted by Farmaha et al.^51^ reported higher soybean yield in a corn-corn-soybean rotation than in a soybean-corn-soybean rotation across a large number of irrigated fields. The results from our study expanded the conclusion that two or more previous years of corn crop sequences resulted in increased soybean yield as compared to one previous year of corn crop sequences. These trials were well-managed, with adequate fertilizer provided, and we do not believe that differences in chemical or physical properties caused the observed differences in yield. One study reported that the rotation of corn and soybeans had a neutral effect on above-ground biomass^52^. Whiting and Crookston^53^ found that the yield benefit from the rotation of soybean with corn was not due to decreases in the incidence of leaf diseases. It has been speculated that rotation-related increased yields were due to enhanced root function^54–56^, decreased soil pathogenic microorganisms, or parasites affecting root growth^57–60^.

In the same plots we compared in this study, Hoss et al.^61^ concluded that these rotations did not produce significant differences in soil physical and chemical properties, but that effects of crop sequence on soybean yield seemed to be the result of multiple interactive biological components in the soil. Indeed, as we show here, biological properties did have an association with yield, likely because soil microorganisms directly drive the carbon^62,63^ and nitrogen cycles^64^. For example, the present study showed that the labile organic carbon pool measured by POXC was higher in CCC and SCC, both sequences with high proportions of corn in the rotation, which corresponded to higher yields of soybean in those plots.

Based on ANCOM analysis of the bulk soil, an uncultured bacterial taxon under family JG30-KF-AS9 (order *Ktedonobacterales*), was found to be significantly abundant at the Monmouth site with a decreasing order of CCC > SCC > CSC > SCS. Although JG30-KF-AS9 was associated with high-yield rotation groups in our study, their biological significance is not known. Similarly, we identified differentially abundant fungal taxa in bulk soil that are of particular interest. Among those observed as a dominant taxon in SCS plots, included some of the most devastating pathogens of crop plants, such as *Macrophomina phaseolina*^65,66^. Pathogens under genera *Macrophomina* are also known to infect soybean roots causing charcoal rot disease^67^. Similarly, *Corynespora*, a genus mostly composed of plant pathogens^68,69^, was found to be significantly abundant in low-yield SCS plots in decreasing orders of SCS > SCC > CSC > CCC at Monmouth and SCS > CSC > SCC > CCC at Urbana. A fungal pathogen from the genera *Corynespora* is the causal agent of soybean frogeye leaf spot disease^70^. *Mycoarthris*, a genus under order *Helotiales*, was another fungal taxon that was significantly abundant at Monmouth and was associated with high-yield rotation groups. However, its biological significance is not well-studied.

At Urbana, *Ascomycota* belonging to family *Chaetothyriacea* (root-associated) were among the most abundant fungal taxa. Although a majority of the *Chaetothyriacea* genera are saprobes and only a very few are known to be plant pathogens and host specific parasites^71–73^, they were associated with low-yield with a higher abundance in SCS rotation, suggesting it is a pathogen.

Based on both ANCOM and Gneiss analysis, root-associated bacterial family *Micromonosporaceae* under order *Micromonosporales* and phylum *Actinobacteria* was associated with low yield. *Micromonosporaceae* are known to act as plant saprophytes or symbionts that thrive under anaerobic conditions^74^, and they have been reported as pathogenic. Gneiss analysis showed several bacterial and fungal taxa present in higher proportions in high and low-yield rotation groups that were not found to be significantly higher by ANCOM analysis. Among those, two bacterial taxa, *Streptomyces*, which were proportionally higher in low-yield, and *Bradyrhizobium*, which were proportionally higher in high-yield, are particularly interesting. Although mostly symbionts, some *Streptomyces* species are known to produce extracellular hydrolytic enzymes that can break down highly stable organic compounds inaccessible to other microbes, infect living plant cells and cause diseases of roots^75,76^. *Bradyrhizobium* helps plants with nitrogen fixation and P and K solubilization^77,78^, thus affecting overall yield. Based on our data, *Streptomyces* only were associated with roots, whereas *Bradyrhizobium* were also present in bulk soil.

The multivariate CCA analysis revealed that the yield of high-yield rotation groups (CCC and SCC) was associated with certain soil health indicators, specifically, protein and POX-C; while low-yield rotation groups (CSC and SCS) were associated with β-glucosidase. Bacterial taxa JG30-KF-AS9, enriched in the CCC plots, was negatively correlated with β-glucosidase in Monmouth, whereas *Microvirga*, enriched in the low-yield treatment, was positively associated with β-glucosidase in Urbana. Both the *Tumularia* and *Idriella*, enriched in the high yield CCC plots, were positively associated with soil protein in Urbana. Some of the differentially abundant fungal taxa from the bulk soil samples were associated with the soil health indicators. For example, *Mycoarthris* correlated positively with POXC, whereas *Macrophomina* and *Corynespora* correlated negatively with POXC and soil protein in Monmouth. Notably, *Corynespora* also negatively correlated with POXC in Urbana.

In this study, the long-term rotational treatments provided the opportunity to determine the impact of crop sequencing on specific microbial taxa and their relationship with yield. Notably, alpha diversity was not significantly different between treatments, which means continuous corn did not result in a reduction of microbiome richness than the other regimes. Although alpha-diversity analysis of microbes did not indicate differences in species richness between high and low-yield rotation groups, we still saw changes in relative species abundances between the rotation groups based on the separation of clusters with a multidimensional scaling analysis, particularly with regards to the root-associated data. Also, some of the bacterial and fungal taxa were found in higher abundance between the rotation groups based on ANCOM and Gneiss analyses.

Soybeans following 14 years of continuous corn (CCC) yielded significantly more than the other three (SCC, CSC, SCS) crop rotations at both locations. The application of custom crop rotation systems in the field could provide many important benefits enhancing soil C concentration and fertility, improving soil physical properties, providing diverse bacterial and fungal communities, and increasing crop yields. This study provided evidence that soil biological properties, including POXC, protein content, specific bacterial 16S rDNA and fungal ITS sequence relative abundances, are significantly correlated with yield shown by the CCA plots. This finding is particularly important given that measuring chemical and physical properties did not provide an adequate explanation for soybean yields differences following these crop sequences^61^. The results suggest that soybean pathogen populations may be determinants, as well as some uncultured bacterial taxa, which still require efforts in culturing and further characterization. Culturability of bacteria has been greatly improved in recent years, and our finding that adding preparations of bacteria, such as those under the order of JG30-KF-AS9 could perhaps be used as a way to increase soybean yields even in fields where soybeans are grown more frequently than once every three or more years.

Crop rotation and sequencing are management tactics that can increase crop yields. The current study found that differential abundances of bacterial and fungal taxa were related to yield differences in a site-specific manner. The soils at Monmouth (Muscatune silt loam) and Urbana (Flanagan silt loam) both developed under prairie vegetation in similar (loess) parent material, and both are highly productive. Soil organic matter content in the top 30 cm was 3.6% at Monmouth and 3.7% at Urbana. Tillage, pesticide application, crop cultivars, and fertilizer usage were similar between the two sites, but averaged over the twelve years before sampling, yields were higher at Monmouth (13.5 t/ha for corn, 4.6 t/ha for soybean) than at Urbana (11.1 and 3.8 t/ha) (E. Nafziger, unpublished data). We believe that yield differences were partially due to weather differences between the two sites which can be found at https://www.isws.illinois.edu/warm/weather/, so it is likely that accumulative weather differences also contributed to differences in microbial composition over time to some degree. Multivariate analysis results indicated that soil- and root-associated microbiome members contributed towards some of the observed yield differences that correlated well with different indicators of soil health. Pathogens, as expected, are associated with low yield, and correlated negatively with soil protein and POXC, whereas taxa selected by the high yield treatment had a positive correlation with soil protein and POXC.

## Methods

### Fields descriptions and original source of the soil and soybean root samples

Field experiments were described in a previously published article^61^.The Urbana site was on Flanagan silt loam soil, and the Monmouth site on Muscatune silt loam; both are highly productive soils. Soil test values (0–15 cm) in fall 2014 were available P 17.6 mg kg^−1^, available K 218 mg kg^−1^, pH 6.6, and soil organic matter (SOM) 3.82% at Monmouth; and available P 21.5 mg kg^−1^, available K 214 mg kg^−1^, pH 5.9, and SOM 3.72% at Urbana. Soils were sampled in July 2016 at a 0–15 cm depth from rotation plots in place for the previous 12–14 years at the University of Illinois Crop Sciences Research Center at Monmouth IL (40.931–90.722) and at Urbana IL (40.048–88.232). The experiment had 4 prior rotation treatments: T1: Continuous corn (CCCCCCCCCCCCCC-S), T2: 2 year of corn-1 year of soybean (SCCSCCSCCSCC-S), T3: 1 year of corn-1 year of soybean (SCSCSCSCSCSC-S) and T4: 1 year of soybean-1 year of corn (CSCSCSCSCSCS-S) ahead of soybean × 4 rep (block) × 3 subsamples per plot in 2016. The field layout followed a random complete block design at both locations. Soybean cultivars P34T07 and P35T58R, both from Pioneer (DuPont Pioneer, Johnston, Iowa) were planted at seeding rates of 355,000 and 395,000 ha^−1^ on 9 May and 16 May at Monmouth and Urbana, respectively. Soybean harvest was done using a plot combine, and yields were corrected to 13% moisture.

At V6 growth stage, soybean roots were sampled from the corresponding plots (with identical prior crop rotation treatments) in July 2017. All experimental research and field studies on the rotation sequences, including the collection of soil samples and soybean root tissues comply with University of Illinois experimental station guidelines. Samples were kept cool during transportation on ice and stored in a − 80 °C horizontal freezer immediately until further processing. Subsamples were taken for DNA extraction in 2 ml microtubes, and a subsample of about 200 g were transferred to a brown paper bag for drying on a greenhouse bench. The soil samples were broken up by a rolling pin and sieved to pass a 2 mm sieve. During processing, the roots were kept on dry ice to prevent microbiome changes.

### Determination of permanganate oxidizable carbon (POXC)

The procedure defined by Weil et al.^10^ was followed for the measurement of POXC. The colorimetric method was used to measure the absorbance by a microplate reader (BioTek Synergy 2 Multi-Mode Microplate Reader) at the wavelength of 550 nm. Samples exceeding the range of the standard curve were diluted with water.

### ACE soil protein index

Soil protein content was measured in triplicate for each soil sample by following a protocol modified from Wright and Upadhyaya and Moebius-Clune et al.^15,79^ by autoclaving (121 °C @ 15 psi for 30 min) a citric acid (pH 7.0) soil extraction and measuring protein in the extract by a colorimetric method (Pierce BCA Protein Assay Kit, Thermo Scientific). Protein assays were performed in 96 well plates incubated at 60 °C for 30 min. After the incubation, the microplate reader (BioTek Synergy 2 Multi-Mode Microplate Reader) was used to obtain the optical density reading at the wavelength of 562 nm.

### Soil β-glucosidase enzyme activity

Soil β-glucosidase enzyme activity was assayed according to the method described by Deng and Tabatabai (1994)^80^. THAM buffer (pH 10) was used to dilute samples at the rate of 1:4 (note: the controls were not diluted) and samples were pipetted into 96 well microplates. The absorbance was measured using a microplate reader (BioTek Synergy 2 Multi-Mode Microplate Reader) at the wavelength of 405 nm.

### Soil and root DNA extraction

The FastDNA Spin Kit (For soil, Cat.No.116560200, MP Biomedicals, Solon, Ohio) was used following the manufacturer’s protocol for DNA extraction from soil with some minor modification. Samples were kept at − 20 °C until further hexadecyltrimethylammonium bromide (CTAB) purification of DNA for post-extraction cleanup^81^. Root DNA was extracted by pulverizing 0.2 g of root that was cut into small pieces (< 0.5 cm) and dry with speed-vac for 4–6 h in bead beating tubes containing two stainless steel BB gun beads. The tubes were dipped in liquid nitrogen and ground for 10 s and return to the liquid nitrogen and ground again repeated until the roots were ground to fine powder. In the tube, 490 μl of water, 20 μl of SDS, and 20 μl of EDTA were added. The tubes were then vortexed vigorously with the horizontal as well as the vertical vortex adapters. The tubes were incubated in a heat block at 68 °C for 10 min and centrifuged at 13,000 rpm for 8 min to pellet cell debris. The supernatant was transferred to a new tube and 30 μl cold potassium acetate was added to each tube, and incubated on ice for 10 min, and then subjected to centrifugation at 13,000 rpm for 8 min. The supernatant was transferred to a new tube and cleaned up using a genomic DNA clean and concentrator-5 kit (Zymo Research, Irvine, CA).

### Bacterial and fungal DNA amplicon sequencing

Bacterial 16S rDNA and fungal nuclear ribosomal internal transcribed spacer (ITS) classifications were amplified and sequenced by University of Minnesota Genomic Center (Minneapolis, Minnesota, USA) using MiSeq-V3 chemistry from a published protocol with a dual-index approach^82^. The 16S V3-V4 and ITS-1 regions were targeted for the bacterial community and fungal community, respectively. Data was deposited in NCBI BioProject SRA accession: PRJNA521547.

### Data analyses

Sample details can be found in Table S1, and soil health indicator data can be found in Table S2. Statistical analyses were performed using R statistical software^83^. Yield, POXC, protein index, and β-glucosidase activities were analyzed by fitting a mixed-effect model with block as a random effect using ‘lme4’^84^ followed by the posthoc tests of LSMeans Differences with ‘emmeans’ packages in R. The following assumptions for linear mixed models were also tested: that errors are linear, independent, normally distributed and homogeneity of variance. The threshold was designated for probability at *P* < 0.05. The classifications of bacteria and fungi were determined using QIIME2^85^, sequences were denoised and filtered using DADA2^86^, and resulting feature tables were then rarefied to perfom core diversity analysis, followed by analyses to detect differential abundance of taxa with the ANCOM^87^ and Gneiss^88^ tests. Taxonomic assignment of representative sequences of fungi and bacteria were performed based on the trained ITS and 16S RNA OTUs clustered at 99% similarities within Unite (version 8)^89^ and Silva132^90^ databases, respectively, using the Naïve-Bayes classifiers^91^ built in QIIME2. Kruskal–Wallis^92^ tests followed by Wilcoxon post hoc tests (where *p* values were significant [*P* < 0.05] for omnibus Kruskal–Wallis tests) were performed to determine significant alpha diversity metrics between the rotation types. Rarefaction curves were generated from multiple samplings of the same sample data with QIIME2 adjusting for variation in the sequencing depth. Canonical correspondance analysis (CCA) was performed using the ‘vegan’ package in R. Data was summarized in the supplementary file, named as CCA. High throughput sequencing data has been submitted to NCBI SRA with the accession number available upon revision or the acceptance of the manuscript.

## Supplementary Information

Supplementary Figures and Tables

## Data Availability

Sequence data is publicly available under NCBI BioProject *SRA accession:* PRJNA521547.

## Abbreviations

ANCOM: Analysis of composition of microbiomes
CCC: Continuous corn
SCC: 2-Yr of corn in a 3-year rotation
CSC: 1-Yr of corn in a 2-yr rotation
SCS: 1-Yr of soybean in a 2-yr rotation
POXC: Permanganate-oxidizable carbon
SOC: Soil organic carbon
SOM: Soil organic matter
