# Supplementary material for: Long term crop rotation effect on subsequent soybean yield explained by soil and root-associated microbiomes and soil health indicators
Source: Sci Rep. 2021 Apr 28;11:9200. doi: 10.1038/s41598-021-88784-6 (PMC8080707; doi:10.1038/s41598-021-88784-6)
Supplement: Supplementary file 1 — Supplementary Figures and Tables [file 41598_2021_88784_MOESM1_ESM.pdf]

Long term crop rotation effect on subsequent soybean yield explained by soil and root-associated microbiomes and soil health indicators

Achal Neupane[1+], Izzet Bulbul[2+], Ziyi Wang[1], R. Michael Lehman[2,3], Emerson Nafziger[4], Shin-Yi Lee Marzano[1,2,5*]

[1]Department of Biology and Microbiology, South Dakota State University, Brookings, SD, 57007, USA.

[2]Department of Agronomy, Horticulture, and Plant Science, South Dakota State University, Brookings, SD, 57007, USA.

[3]United States Department of Agriculture, Agricultural Research Service, North Central Agricultural Research Laboratory, Brookings, SD, 57006, USA.

[4]Department of Crop Sciences, University of Illinois, Urbana, IL, 61801, USA.

[5]Present Address: United States Department of Agriculture, Agricultural Research Service, Application Technology Research Unit, Toledo, OH ,43606, USA.

[+]Contribute equally.

*Correspondence should be addressed to S.L.M. (email: shinyi.marzano@usda.gov)

Figure S1. Alpha diversity estimated by rarefaction curves. Bacterial (A) and fungal (B) diversities at the Monmouth site, and bacterial (C) and fungal (D) at the Urbana site associated with bulk soil; bacterial (E) and fungal (F) at the Monmouth site, and bacterial (G) and fungal (H) at the Urbana site associated with roots are shown.

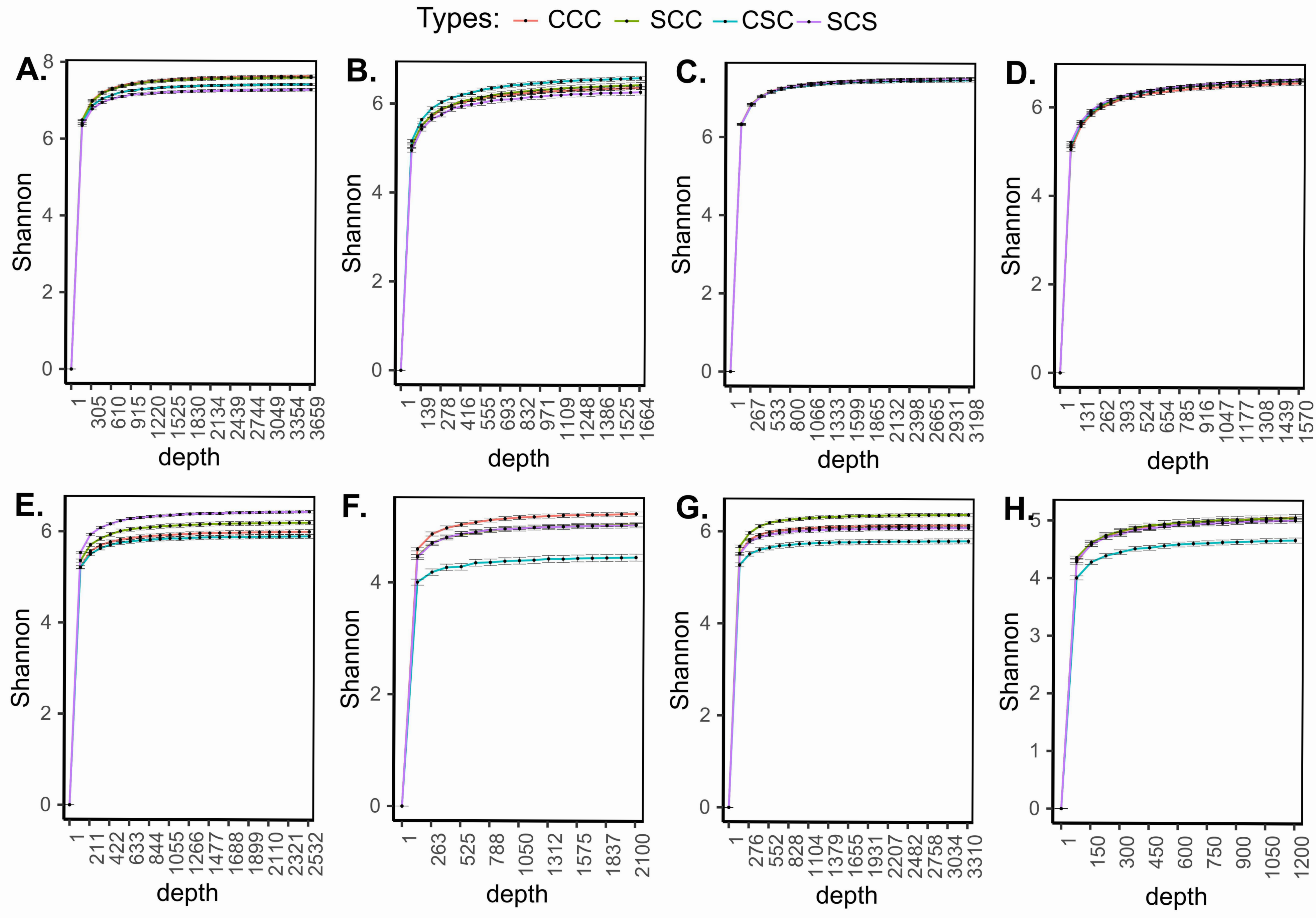
Types: CCC SCC CSC SCS
A.
B.
C.
D.
E.
F.
G.
H.
Shannon
depth

Figure S2. Beta-diversity visualized by EMPeror. Bacterial (A) and fungal (B) diversities at the Monmouth site, and bacterial (C) and fungal (D) diversities at the Urbana site associated with bulk soil; bacterial (E) and fungal (F) diversities at the Monmouth site, and bacterial (G) and fungal (H) diversities at the Urbana site associated with roots are shown.

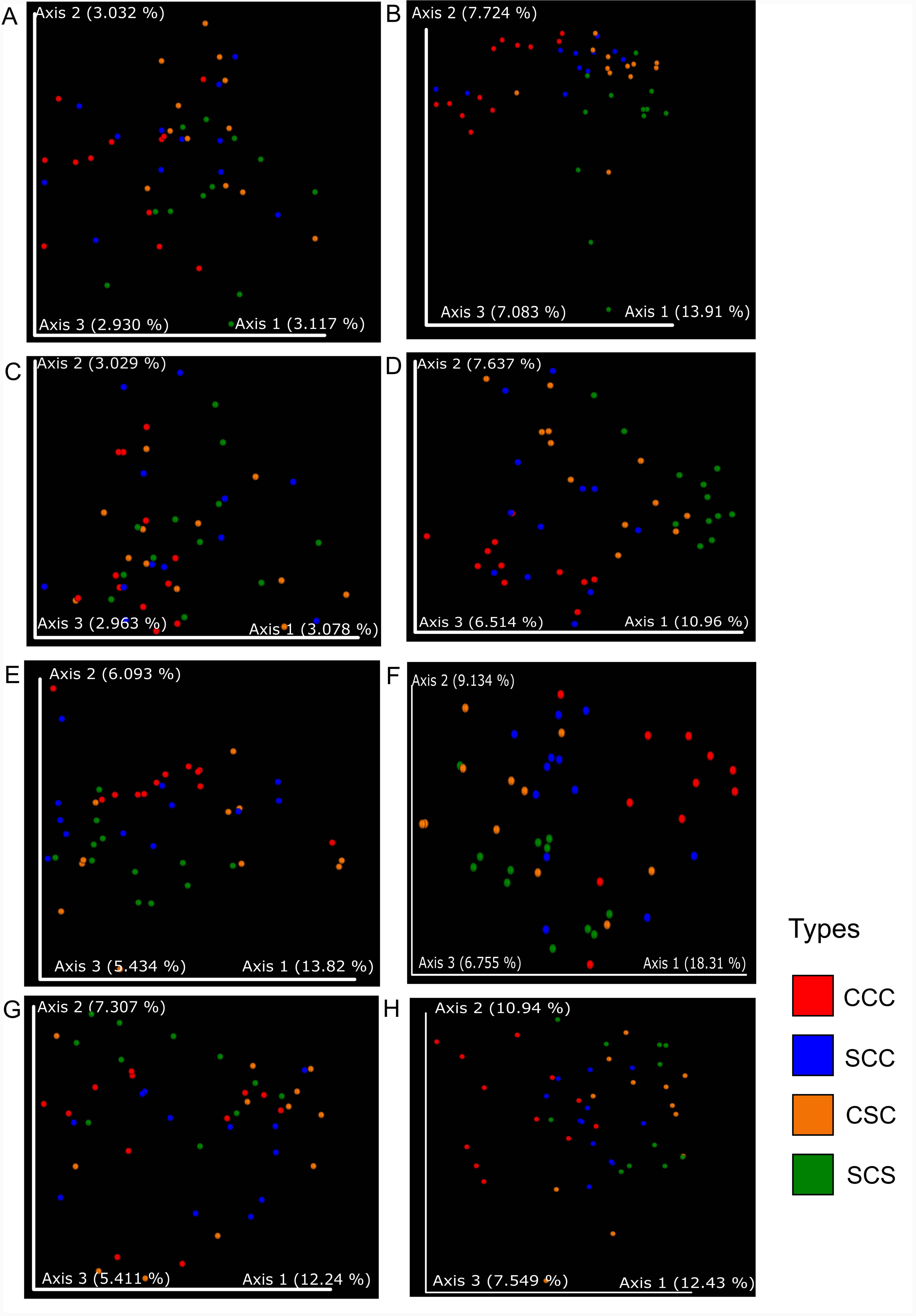
A
Axis 2 (3.032 %)
Axis 3 (2.930 %)
Axis 1 (3.117 %)
B
Axis 2 (7.724 %)
Axis 3 (7.083 %)
Axis 1 (13.91 %)
C
Axis 2 (3.029 %)
Axis 3 (2.963 %)
Axis 1 (3.078 %)
D
Axis 2 (7.637 %)
Axis 3 (6.514 %)
Axis 1 (10.96 %)
E
Axis 2 (6.093 %)
Axis 3 (5.434 %)
Axis 1 (13.82 %)
F
Axis 2 (9.134 %)
Axis 3 (6.755 %)
Axis 1 (18.31 %)
G
Axis 2 (7.307 %)
Axis 3 (5.411 %)
Axis 1 (12.24 %)
H
Axis 2 (10.94 %)
Axis 3 (7.549 %)
Axis 1 (12.43 %)
Types
CCC
SCC
CSC
SCS

Table S1 Sample details.

SampleID	Rotation	Location	Source	Data_type
M_T1_R1_S1_16S_S50	CCC	Monmouth	Soil	16S
M_T1_R1_S2_16S_S62	CCC	Monmouth	Soil	16S
M_T1_R1_S3_16S_S74	CCC	Monmouth	Soil	16S
M_T1_R2_S1_16S_S86	CCC	Monmouth	Soil	16S
M_T1_R2_S2_16S_S3	CCC	Monmouth	Soil	16S
M_T1_R2_S3_16S_S15	CCC	Monmouth	Soil	16S
M_T1_R3_S1_16S_S27	CCC	Monmouth	Soil	16S
M_T1_R3_S2_16S_S39	CCC	Monmouth	Soil	16S
M_T1_R3_S3_16S_S51	CCC	Monmouth	Soil	16S
M_T1_R4_S1_16S_S63	CCC	Monmouth	Soil	16S
M_T1_R4_S2_16S_S75	CCC	Monmouth	Soil	16S
M_T1_R4_S3_16S_S87	CCC	Monmouth	Soil	16S
M_T2_R1_S1_16S_S53	SCC	Monmouth	Soil	16S
M_T2_R1_S2_16S_S65	SCC	Monmouth	Soil	16S
M_T2_R1_S3_16S_S77	SCC	Monmouth	Soil	16S
M_T2_R2_S1_16S_S89	SCC	Monmouth	Soil	16S
M_T2_R2_S2_16S_S6	SCC	Monmouth	Soil	16S
M_T2_R2_S3_16S_S18	SCC	Monmouth	Soil	16S
M_T2_R3_S1_16S_S30	SCC	Monmouth	Soil	16S
M_T2_R3_S2_16S_S42	SCC	Monmouth	Soil	16S
M_T2_R3_S3_16S_S54	SCC	Monmouth	Soil	16S
M_T2_R4_S1_16S_S66	SCC	Monmouth	Soil	16S
M_T2_R4_S2_16S_S78	SCC	Monmouth	Soil	16S
M_T2_R4_S3_16S_S90	SCC	Monmouth	Soil	16S
M_T3_R1_S1_16S_S56	CSC	Monmouth	Soil	16S
M_T3_R1_S2_16S_S68	CSC	Monmouth	Soil	16S
M_T3_R1_S3_16S_S80	CSC	Monmouth	Soil	16S
M_T3_R2_S1_16S_S92	CSC	Monmouth	Soil	16S
M_T3_R2_S2_16S_S9	CSC	Monmouth	Soil	16S
M_T3_R2_S3_16S_S21	CSC	Monmouth	Soil	16S
M_T3_R3_S1_16S_S33	CSC	Monmouth	Soil	16S
M_T3_R3_S2_16S_S45	CSC	Monmouth	Soil	16S
M_T3_R3_S3_16S_S57	CSC	Monmouth	Soil	16S
M_T3_R4_S1_16S_S69	CSC	Monmouth	Soil	16S
M_T3_R4_S2_16S_S81	CSC	Monmouth	Soil	16S
M_T3_R4_S3_16S_S93	CSC	Monmouth	Soil	16S
M_T4_R1_S1_16S_S59	SCS	Monmouth	Soil	16S
M_T4_R1_S2_16S_S71	SCS	Monmouth	Soil	16S
M_T4_R1_S3_16S_S83	SCS	Monmouth	Soil	16S
M_T4_R2_S1_16S_S95	SCS	Monmouth	Soil	16S

M_T4_R2_S2_16S_S12	SCS	Monmouth	Soil	16S
M_T4_R2_S3_16S_S24	SCS	Monmouth	Soil	16S
M_T4_R3_S1_16S_S36	SCS	Monmouth	Soil	16S
M_T4_R3_S2_16S_S48	SCS	Monmouth	Soil	16S
M_T4_R3_S3_16S_S60	SCS	Monmouth	Soil	16S
M_T4_R4_S1_16S_S72	SCS	Monmouth	Soil	16S
M_T4_R4_S2_16S_S193	SCS	Monmouth	Soil	16S
M_T4_R4_S3_16S_S203	SCS	Monmouth	Soil	16S
U_T1_R1_S1_16S_S1	CCC	Urbana	Soil	16S
U_T1_R1_S2_16S_S13	CCC	Urbana	Soil	16S
U_T1_R1_S3_16S_S25	CCC	Urbana	Soil	16S
U_T1_R2_S1_16S_S37	CCC	Urbana	Soil	16S
U_T1_R2_S2_16S_S49	CCC	Urbana	Soil	16S
U_T1_R2_S3_16S_S61	CCC	Urbana	Soil	16S
U_T1_R3_S1_16S_S73	CCC	Urbana	Soil	16S
U_T1_R3_S2_16S_S85	CCC	Urbana	Soil	16S
U_T1_R3_S3_16S_S2	CCC	Urbana	Soil	16S
U_T1_R4_S1_16S_S14	CCC	Urbana	Soil	16S
U_T1_R4_S2_16S_S26	CCC	Urbana	Soil	16S
U_T1_R4_S3_16S_S38	CCC	Urbana	Soil	16S
U_T2_R1_S1_16S_S4	SCC	Urbana	Soil	16S
U_T2_R1_S2_16S_S16	SCC	Urbana	Soil	16S
U_T2_R1_S3_16S_S28	SCC	Urbana	Soil	16S
U_T2_R2_S1_16S_S40	SCC	Urbana	Soil	16S
U_T2_R2_S2_16S_S52	SCC	Urbana	Soil	16S
U_T2_R2_S3_16S_S64	SCC	Urbana	Soil	16S
U_T2_R3_S1_16S_S76	SCC	Urbana	Soil	16S
U_T2_R3_S2_16S_S88	SCC	Urbana	Soil	16S
U_T2_R3_S3_16S_S5	SCC	Urbana	Soil	16S
U_T2_R4_S1_16S_S17	SCC	Urbana	Soil	16S
U_T2_R4_S2_16S_S29	SCC	Urbana	Soil	16S
U_T2_R4_S3_16S_S41	SCC	Urbana	Soil	16S
U_T3_R1_S1_16S_S7	CSC	Urbana	Soil	16S
U_T3_R1_S2_16S_S19	CSC	Urbana	Soil	16S
U_T3_R1_S3_16S_S31	CSC	Urbana	Soil	16S
U_T3_R2_S1_16S_S43	CSC	Urbana	Soil	16S
U_T3_R2_S2_16S_S55	CSC	Urbana	Soil	16S
U_T3_R2_S3_16S_S67	CSC	Urbana	Soil	16S
U_T3_R3_S1_16S_S79	CSC	Urbana	Soil	16S
U_T3_R3_S2_16S_S91	CSC	Urbana	Soil	16S
U_T3_R3_S3_16S_S8	CSC	Urbana	Soil	16S

U_T3_R4_S1_16S_S20	CSC	Urbana	Soil	16S
U_T3_R4_S2_16S_S32	CSC	Urbana	Soil	16S
U_T3_R4_S3_16S_S44	CSC	Urbana	Soil	16S
U_T4_R1_S1_16S_S10	SCS	Urbana	Soil	16S
U_T4_R1_S2_16S_S22	SCS	Urbana	Soil	16S
U_T4_R1_S3_16S_S34	SCS	Urbana	Soil	16S
U_T4_R2_S1_16S_S46	SCS	Urbana	Soil	16S
U_T4_R2_S2_16S_S58	SCS	Urbana	Soil	16S
U_T4_R2_S3_16S_S70	SCS	Urbana	Soil	16S
U_T4_R3_S1_16S_S82	SCS	Urbana	Soil	16S
U_T4_R3_S2_16S_S94	SCS	Urbana	Soil	16S
U_T4_R3_S3_16S_S11	SCS	Urbana	Soil	16S
U_T4_R4_S1_16S_S23	SCS	Urbana	Soil	16S
U_T4_R4_S2_16S_S35	SCS	Urbana	Soil	16S
U_T4_R4_S3_16S_S47	SCS	Urbana	Soil	16S
M_T1_R1_S1_ITS_S146	CCC	Monmouth	Soil	ITS
M_T1_R1_S2_ITS_S158	CCC	Monmouth	Soil	ITS
M_T1_R1_S3_ITS_S170	CCC	Monmouth	Soil	ITS
M_T1_R2_S1_ITS_S182	CCC	Monmouth	Soil	ITS
M_T1_R2_S2_ITS_S99	CCC	Monmouth	Soil	ITS
M_T1_R2_S3_ITS_S111	CCC	Monmouth	Soil	ITS
M_T1_R3_S1_ITS_S123	CCC	Monmouth	Soil	ITS
M_T1_R3_S2_ITS_S135	CCC	Monmouth	Soil	ITS
M_T1_R3_S3_ITS_S147	CCC	Monmouth	Soil	ITS
M_T1_R4_S1_ITS_S159	CCC	Monmouth	Soil	ITS
M_T1_R4_S2_ITS_S171	CCC	Monmouth	Soil	ITS
M_T1_R4_S3_ITS_S183	CCC	Monmouth	Soil	ITS
M_T2_R1_S1_ITS_S149	SCC	Monmouth	Soil	ITS
M_T2_R1_S2_ITS_S161	SCC	Monmouth	Soil	ITS
M_T2_R1_S3_ITS_S173	SCC	Monmouth	Soil	ITS
M_T2_R2_S1_ITS_S185	SCC	Monmouth	Soil	ITS
M_T2_R2_S2_ITS_S102	SCC	Monmouth	Soil	ITS
M_T2_R2_S3_ITS_S114	SCC	Monmouth	Soil	ITS
M_T2_R3_S1_ITS_S126	SCC	Monmouth	Soil	ITS
M_T2_R3_S2_ITS_S138	SCC	Monmouth	Soil	ITS
M_T2_R3_S3_ITS_S150	SCC	Monmouth	Soil	ITS
M_T2_R4_S1_ITS_S162	SCC	Monmouth	Soil	ITS
M_T2_R4_S2_ITS_S174	SCC	Monmouth	Soil	ITS
M_T2_R4_S3_ITS_S186	SCC	Monmouth	Soil	ITS
M_T3_R1_S1_ITS_S152	CSC	Monmouth	Soil	ITS
M_T3_R1_S2_ITS_S164	CSC	Monmouth	Soil	ITS

M_T3_R1_S3_ITS_S176	CSC	Monmouth	Soil	ITS
M_T3_R2_S1_ITS_S188	CSC	Monmouth	Soil	ITS
M_T3_R2_S2_ITS_S105	CSC	Monmouth	Soil	ITS
M_T3_R2_S3_ITS_S117	CSC	Monmouth	Soil	ITS
M_T3_R3_S1_ITS_S129	CSC	Monmouth	Soil	ITS
M_T3_R3_S2_ITS_S141	CSC	Monmouth	Soil	ITS
M_T3_R3_S3_ITS_S153	CSC	Monmouth	Soil	ITS
M_T3_R4_S1_ITS_S165	CSC	Monmouth	Soil	ITS
M_T3_R4_S2_ITS_S177	CSC	Monmouth	Soil	ITS
M_T3_R4_S3_ITS_S189	CSC	Monmouth	Soil	ITS
M_T4_R1_S1_ITS_S155	SCS	Monmouth	Soil	ITS
M_T4_R1_S2_ITS_S167	SCS	Monmouth	Soil	ITS
M_T4_R1_S3_ITS_S179	SCS	Monmouth	Soil	ITS
M_T4_R2_S1_ITS_S191	SCS	Monmouth	Soil	ITS
M_T4_R2_S2_ITS_S108	SCS	Monmouth	Soil	ITS
M_T4_R2_S3_ITS_S120	SCS	Monmouth	Soil	ITS
M_T4_R3_S1_ITS_S132	SCS	Monmouth	Soil	ITS
M_T4_R3_S2_ITS_S144	SCS	Monmouth	Soil	ITS
M_T4_R3_S3_ITS_S156	SCS	Monmouth	Soil	ITS
M_T4_R4_S1_ITS_S168	SCS	Monmouth	Soil	ITS
M_T4_R4_S2_ITS_S263	SCS	Monmouth	Soil	ITS
M_T4_R4_S3_ITS_S265	SCS	Monmouth	Soil	ITS
U_T1_R1_S1_ITS_S97	CCC	Urbana	Soil	ITS
U_T1_R1_S2_ITS_S109	CCC	Urbana	Soil	ITS
U_T1_R1_S3_ITS_S121	CCC	Urbana	Soil	ITS
U_T1_R2_S1_ITS_S133	CCC	Urbana	Soil	ITS
U_T1_R2_S2_ITS_S145	CCC	Urbana	Soil	ITS
U_T1_R2_S3_ITS_S157	CCC	Urbana	Soil	ITS
U_T1_R3_S1_ITS_S169	CCC	Urbana	Soil	ITS
U_T1_R3_S2_ITS_S181	CCC	Urbana	Soil	ITS
U_T1_R3_S3_ITS_S98	CCC	Urbana	Soil	ITS
U_T1_R4_S1_ITS_S110	CCC	Urbana	Soil	ITS
U_T1_R4_S2_ITS_S122	CCC	Urbana	Soil	ITS
U_T1_R4_S3_ITS_S134	CCC	Urbana	Soil	ITS
U_T2_R1_S1_ITS_S100	SCC	Urbana	Soil	ITS
U_T2_R1_S2_ITS_S112	SCC	Urbana	Soil	ITS
U_T2_R1_S3_ITS_S124	SCC	Urbana	Soil	ITS
U_T2_R2_S1_ITS_S136	SCC	Urbana	Soil	ITS
U_T2_R2_S2_ITS_S148	SCC	Urbana	Soil	ITS
U_T2_R2_S3_ITS_S160	SCC	Urbana	Soil	ITS
U_T2_R3_S1_ITS_S172	SCC	Urbana	Soil	ITS

U_T2_R3_S2_ITS_S184	SCC	Urbana	Soil	ITS
U_T2_R3_S3_ITS_S101	SCC	Urbana	Soil	ITS
U_T2_R4_S1_ITS_S113	SCC	Urbana	Soil	ITS
U_T2_R4_S2_ITS_S125	SCC	Urbana	Soil	ITS
U_T2_R4_S3_ITS_S137	SCC	Urbana	Soil	ITS
U_T3_R1_S1_ITS_S103	CSC	Urbana	Soil	ITS
U_T3_R1_S2_ITS_S115	CSC	Urbana	Soil	ITS
U_T3_R1_S3_ITS_S127	CSC	Urbana	Soil	ITS
U_T3_R2_S1_ITS_S139	CSC	Urbana	Soil	ITS
U_T3_R2_S2_ITS_S151	CSC	Urbana	Soil	ITS
U_T3_R2_S3_ITS_S163	CSC	Urbana	Soil	ITS
U_T3_R3_S1_ITS_S175	CSC	Urbana	Soil	ITS
U_T3_R3_S2_ITS_S187	CSC	Urbana	Soil	ITS
U_T3_R3_S3_ITS_S104	CSC	Urbana	Soil	ITS
U_T3_R4_S1_ITS_S116	CSC	Urbana	Soil	ITS
U_T3_R4_S2_ITS_S128	CSC	Urbana	Soil	ITS
U_T3_R4_S3_ITS_S140	CSC	Urbana	Soil	ITS
U_T4_R1_S1_ITS_S106	SCS	Urbana	Soil	ITS
U_T4_R1_S2_ITS_S118	SCS	Urbana	Soil	ITS
U_T4_R1_S3_ITS_S130	SCS	Urbana	Soil	ITS
U_T4_R2_S1_ITS_S142	SCS	Urbana	Soil	ITS
U_T4_R2_S2_ITS_S154	SCS	Urbana	Soil	ITS
U_T4_R2_S3_ITS_S166	SCS	Urbana	Soil	ITS
U_T4_R3_S1_ITS_S178	SCS	Urbana	Soil	ITS
U_T4_R3_S2_ITS_S190	SCS	Urbana	Soil	ITS
U_T4_R3_S3_ITS_S107	SCS	Urbana	Soil	ITS
U_T4_R4_S1_ITS_S119	SCS	Urbana	Soil	ITS
U_T4_R4_S2_ITS_S131	SCS	Urbana	Soil	ITS
U_T4_R4_S3_ITS_S143	SCS	Urbana	Soil	ITS
16S_M_T1_R1_S1_S50	CCC	Monmouth	Root	16S
16S_M_T1_R1_S2_S62	CCC	Monmouth	Root	16S
16S_M_T1_R1_S3_S74	CCC	Monmouth	Root	16S
16S_M_T1_R2_S1_S85	CCC	Monmouth	Root	16S
16S_M_T1_R2_S2_S3	CCC	Monmouth	Root	16S
16S_M_T1_R2_S3_S15	CCC	Monmouth	Root	16S
16S_M_T1_R3_S1_S27	CCC	Monmouth	Root	16S
16S_M_T1_R3_S2_S39	CCC	Monmouth	Root	16S
16S_M_T1_R3_S3_S51	CCC	Monmouth	Root	16S
16S_M_T1_R4_S1_S63	CCC	Monmouth	Root	16S
16S_M_T1_R4_S2_S75	CCC	Monmouth	Root	16S
16S_M_T1_R4_S3_S86	CCC	Monmouth	Root	16S

16S_M_T2_R1_S1_S53	SCC	Monmouth	Root	16S
16S_M_T2_R1_S2_S65	SCC	Monmouth	Root	16S
16S_M_T2_R1_S3_S77	SCC	Monmouth	Root	16S
16S_M_T2_R2_S1_S88	SCC	Monmouth	Root	16S
16S_M_T2_R2_S2_S6	SCC	Monmouth	Root	16S
16S_M_T2_R2_S3_S18	SCC	Monmouth	Root	16S
16S_M_T2_R3_S1_S30	SCC	Monmouth	Root	16S
16S_M_T2_R3_S2_S42	SCC	Monmouth	Root	16S
16S_M_T2_R3_S3_S54	SCC	Monmouth	Root	16S
16S_M_T2_R4_S1_S66	SCC	Monmouth	Root	16S
16S_M_T2_R4_S2_S78	SCC	Monmouth	Root	16S
16S_M_T2_R4_S3_S89	SCC	Monmouth	Root	16S
16S_M_T3_R1_S1_S56	CSC	Monmouth	Root	16S
16S_M_T3_R1_S2_S68	CSC	Monmouth	Root	16S
16S_M_T3_R1_S3_S80	CSC	Monmouth	Root	16S
16S_M_T3_R2_S1_S91	CSC	Monmouth	Root	16S
16S_M_T3_R2_S2_S9	CSC	Monmouth	Root	16S
16S_M_T3_R2_S3_S21	CSC	Monmouth	Root	16S
16S_M_T3_R3_S1_S33	CSC	Monmouth	Root	16S
16S_M_T3_R3_S2_S45	CSC	Monmouth	Root	16S
16S_M_T3_R3_S3_S57	CSC	Monmouth	Root	16S
16S_M_T3_R4_S1_S69	CSC	Monmouth	Root	16S
16S_M_T3_R4_S2_S81	CSC	Monmouth	Root	16S
16S_M_T3_R4_S3_S92	CSC	Monmouth	Root	16S
16S_M_T4_R1_S1_S59	SCS	Monmouth	Root	16S
16S_M_T4_R1_S2_S71	SCS	Monmouth	Root	16S
16S_M_T4_R1_S3_S83	SCS	Monmouth	Root	16S
16S_M_T4_R2_S1_S94	SCS	Monmouth	Root	16S
16S_M_T4_R2_S2_S12	SCS	Monmouth	Root	16S
16S_M_T4_R2_S3_S24	SCS	Monmouth	Root	16S
16S_M_T4_R3_S1_S36	SCS	Monmouth	Root	16S
16S_M_T4_R3_S2_S48	SCS	Monmouth	Root	16S
16S_M_T4_R3_S3_S60	SCS	Monmouth	Root	16S
16S_M_T4_R4_S1_S72	SCS	Monmouth	Root	16S
16S_M_T4_R4_S2_S95	SCS	Monmouth	Root	16S
16S_M_T4_R4_S3_S104	SCS	Monmouth	Root	16S
16S_U_T1_R1_S1_S1	CCC	Urbana	Root	16S
16S_U_T1_R1_S2_S13	CCC	Urbana	Root	16S
16S_U_T1_R1_S3_S25	CCC	Urbana	Root	16S
16S_U_T1_R2_S1_S37	CCC	Urbana	Root	16S
16S_U_T1_R2_S2_S49	CCC	Urbana	Root	16S

16S_U_T1_R2_S3_S61	CCC	Urbana	Root	16S
16S_U_T1_R3_S1_S73	CCC	Urbana	Root	16S
16S_U_T1_R3_S2_S84	CCC	Urbana	Root	16S
16S_U_T1_R3_S3_S2	CCC	Urbana	Root	16S
16S_U_T1_R4_S1_S14	CCC	Urbana	Root	16S
16S_U_T1_R4_S2_S26	CCC	Urbana	Root	16S
16S_U_T1_R4_S3_S38	CCC	Urbana	Root	16S
16S_U_T2_R1_S1_S4	SCC	Urbana	Root	16S
16S_U_T2_R1_S2_S16	SCC	Urbana	Root	16S
16S_U_T2_R1_S3_S28	SCC	Urbana	Root	16S
16S_U_T2_R2_S1_S40	SCC	Urbana	Root	16S
16S_U_T2_R2_S2_S52	SCC	Urbana	Root	16S
16S_U_T2_R2_S3_S64	SCC	Urbana	Root	16S
16S_U_T2_R3_S1_S76	SCC	Urbana	Root	16S
16S_U_T2_R3_S2_S87	SCC	Urbana	Root	16S
16S_U_T2_R3_S3_S5	SCC	Urbana	Root	16S
16S_U_T2_R4_S1_S17	SCC	Urbana	Root	16S
16S_U_T2_R4_S2_S29	SCC	Urbana	Root	16S
16S_U_T2_R4_S3_S41	SCC	Urbana	Root	16S
16S_U_T3_R1_S1_S7	CSC	Urbana	Root	16S
16S_U_T3_R1_S2_S19	CSC	Urbana	Root	16S
16S_U_T3_R1_S3_S31	CSC	Urbana	Root	16S
16S_U_T3_R2_S1_S43	CSC	Urbana	Root	16S
16S_U_T3_R2_S2_S55	CSC	Urbana	Root	16S
16S_U_T3_R2_S3_S67	CSC	Urbana	Root	16S
16S_U_T3_R3_S1_S79	CSC	Urbana	Root	16S
16S_U_T3_R3_S2_S90	CSC	Urbana	Root	16S
16S_U_T3_R3_S3_S8	CSC	Urbana	Root	16S
16S_U_T3_R4_S1_S20	CSC	Urbana	Root	16S
16S_U_T3_R4_S2_S32	CSC	Urbana	Root	16S
16S_U_T3_R4_S3_S44	CSC	Urbana	Root	16S
16S_U_T4_R1_S1_S10	SCS	Urbana	Root	16S
16S_U_T4_R1_S2_S22	SCS	Urbana	Root	16S
16S_U_T4_R1_S3_S34	SCS	Urbana	Root	16S
16S_U_T4_R2_S1_S46	SCS	Urbana	Root	16S
16S_U_T4_R2_S2_S58	SCS	Urbana	Root	16S
16S_U_T4_R2_S3_S70	SCS	Urbana	Root	16S
16S_U_T4_R3_S1_S82	SCS	Urbana	Root	16S
16S_U_T4_R3_S2_S93	SCS	Urbana	Root	16S
16S_U_T4_R3_S3_S11	SCS	Urbana	Root	16S
16S_U_T4_R4_S1_S23	SCS	Urbana	Root	16S

16S_U_T4_R4_S2_S35	SCS	Urbana	Root	16S
16S_U_T4_R4_S3_S47	SCS	Urbana	Root	16S
ITS_M_T1_R1_S1_S210	CCC	Monmouth	Root	ITS
ITS_M_T1_R1_S2_S222	CCC	Monmouth	Root	ITS
ITS_M_T1_R1_S3_S234	CCC	Monmouth	Root	ITS
ITS_M_T1_R2_S1_S245	CCC	Monmouth	Root	ITS
ITS_M_T1_R2_S2_S163	CCC	Monmouth	Root	ITS
ITS_M_T1_R2_S3_S175	CCC	Monmouth	Root	ITS
ITS_M_T1_R3_S1_S187	CCC	Monmouth	Root	ITS
ITS_M_T1_R3_S2_S199	CCC	Monmouth	Root	ITS
ITS_M_T1_R3_S3_S211	CCC	Monmouth	Root	ITS
ITS_M_T1_R4_S1_S223	CCC	Monmouth	Root	ITS
ITS_M_T1_R4_S2_S235	CCC	Monmouth	Root	ITS
ITS_M_T1_R4_S3_S246	CCC	Monmouth	Root	ITS
ITS_M_T2_R1_S1_S213	SCC	Monmouth	Root	ITS
ITS_M_T2_R1_S2_S225	SCC	Monmouth	Root	ITS
ITS_M_T2_R1_S3_S237	SCC	Monmouth	Root	ITS
ITS_M_T2_R2_S1_S248	SCC	Monmouth	Root	ITS
ITS_M_T2_R2_S2_S166	SCC	Monmouth	Root	ITS
ITS_M_T2_R2_S3_S178	SCC	Monmouth	Root	ITS
ITS_M_T2_R3_S1_S190	SCC	Monmouth	Root	ITS
ITS_M_T2_R3_S2_S202	SCC	Monmouth	Root	ITS
ITS_M_T2_R3_S3_S214	SCC	Monmouth	Root	ITS
ITS_M_T2_R4_S1_S226	SCC	Monmouth	Root	ITS
ITS_M_T2_R4_S2_S238	SCC	Monmouth	Root	ITS
ITS_M_T2_R4_S3_S249	SCC	Monmouth	Root	ITS
ITS_M_T3_R1_S1_S216	CSC	Monmouth	Root	ITS
ITS_M_T3_R1_S2_S228	CSC	Monmouth	Root	ITS
ITS_M_T3_R1_S3_S240	CSC	Monmouth	Root	ITS
ITS_M_T3_R2_S1_S251	CSC	Monmouth	Root	ITS
ITS_M_T3_R2_S2_S169	CSC	Monmouth	Root	ITS
ITS_M_T3_R2_S3_S181	CSC	Monmouth	Root	ITS
ITS_M_T3_R3_S1_S193	CSC	Monmouth	Root	ITS
ITS_M_T3_R3_S2_S205	CSC	Monmouth	Root	ITS
ITS_M_T3_R3_S3_S217	CSC	Monmouth	Root	ITS
ITS_M_T3_R4_S1_S229	CSC	Monmouth	Root	ITS
ITS_M_T3_R4_S2_S241	CSC	Monmouth	Root	ITS
ITS_M_T3_R4_S3_S252	CSC	Monmouth	Root	ITS
ITS_M_T4_R1_S1_S219	SCS	Monmouth	Root	ITS
ITS_M_T4_R1_S2_S231	SCS	Monmouth	Root	ITS
ITS_M_T4_R1_S3_S243	SCS	Monmouth	Root	ITS

ITS_M_T4_R2_S1_S254	SCS	Monmouth	Root	ITS
ITS_M_T4_R2_S2_S172	SCS	Monmouth	Root	ITS
ITS_M_T4_R2_S3_S184	SCS	Monmouth	Root	ITS
ITS_M_T4_R3_S1_S196	SCS	Monmouth	Root	ITS
ITS_M_T4_R3_S2_S208	SCS	Monmouth	Root	ITS
ITS_M_T4_R3_S3_S220	SCS	Monmouth	Root	ITS
ITS_M_T4_R4_S1_S232	SCS	Monmouth	Root	ITS
ITS_M_T4_R4_S2_S255	SCS	Monmouth	Root	ITS
ITS_M_T4_R4_S3_S264	SCS	Monmouth	Root	ITS
ITS_U_T1_R1_S1_S161	CCC	Urbana	Root	ITS
ITS_U_T1_R1_S2_S173	CCC	Urbana	Root	ITS
ITS_U_T1_R1_S3_S185	CCC	Urbana	Root	ITS
ITS_U_T1_R2_S1_S197	CCC	Urbana	Root	ITS
ITS_U_T1_R2_S2_S209	CCC	Urbana	Root	ITS
ITS_U_T1_R2_S3_S221	CCC	Urbana	Root	ITS
ITS_U_T1_R3_S1_S233	CCC	Urbana	Root	ITS
ITS_U_T1_R3_S2_S244	CCC	Urbana	Root	ITS
ITS_U_T1_R3_S3_S162	CCC	Urbana	Root	ITS
ITS_U_T1_R4_S1_S174	CCC	Urbana	Root	ITS
ITS_U_T1_R4_S2_S186	CCC	Urbana	Root	ITS
ITS_U_T1_R4_S3_S198	CCC	Urbana	Root	ITS
ITS_U_T2_R1_S1_S164	SCC	Urbana	Root	ITS
ITS_U_T2_R1_S2_S176	SCC	Urbana	Root	ITS
ITS_U_T2_R1_S3_S188	SCC	Urbana	Root	ITS
ITS_U_T2_R2_S1_S200	SCC	Urbana	Root	ITS
ITS_U_T2_R2_S2_S212	SCC	Urbana	Root	ITS
ITS_U_T2_R2_S3_S224	SCC	Urbana	Root	ITS
ITS_U_T2_R3_S1_S236	SCC	Urbana	Root	ITS
ITS_U_T2_R3_S2_S247	SCC	Urbana	Root	ITS
ITS_U_T2_R3_S3_S165	SCC	Urbana	Root	ITS
ITS_U_T2_R4_S1_S177	SCC	Urbana	Root	ITS
ITS_U_T2_R4_S2_S189	SCC	Urbana	Root	ITS
ITS_U_T2_R4_S3_S201	SCC	Urbana	Root	ITS
ITS_U_T3_R1_S1_S167	CSC	Urbana	Root	ITS
ITS_U_T3_R1_S2_S179	CSC	Urbana	Root	ITS
ITS_U_T3_R1_S3_S191	CSC	Urbana	Root	ITS
ITS_U_T3_R2_S1_S203	CSC	Urbana	Root	ITS
ITS_U_T3_R2_S2_S215	CSC	Urbana	Root	ITS
ITS_U_T3_R2_S3_S227	CSC	Urbana	Root	ITS
ITS_U_T3_R3_S1_S239	CSC	Urbana	Root	ITS
ITS_U_T3_R3_S2_S250	CSC	Urbana	Root	ITS

ITS_U_T3_R3_S3_S168	CSC	Urbana	Root	ITS
ITS_U_T3_R4_S1_S180	CSC	Urbana	Root	ITS
ITS_U_T3_R4_S2_S192	CSC	Urbana	Root	ITS
ITS_U_T3_R4_S3_S204	CSC	Urbana	Root	ITS
ITS_U_T4_R1_S1_S170	SCS	Urbana	Root	ITS
ITS_U_T4_R1_S2_S182	SCS	Urbana	Root	ITS
ITS_U_T4_R1_S3_S194	SCS	Urbana	Root	ITS
ITS_U_T4_R2_S1_S206	SCS	Urbana	Root	ITS
ITS_U_T4_R2_S2_S218	SCS	Urbana	Root	ITS
ITS_U_T4_R2_S3_S230	SCS	Urbana	Root	ITS
ITS_U_T4_R3_S1_S242	SCS	Urbana	Root	ITS
ITS_U_T4_R3_S2_S253	SCS	Urbana	Root	ITS
ITS_U_T4_R3_S3_S171	SCS	Urbana	Root	ITS
ITS_U_T4_R4_S1_S183	SCS	Urbana	Root	ITS
ITS_U_T4_R4_S2_S195	SCS	Urbana	Root	ITS
ITS_U_T4_R4_S3_S207	SCS	Urbana	Root	ITS

Table S2 Soil health indicators data.

Samples	POX_C	B_glucosidase	Protein
M_T1_R1_S1_16S_S50	869.2	1.9	6284.2
M_T1_R1_S2_16S_S62	841.4	1.6	6250.5
M_T1_R1_S3_16S_S74	720.3	1.4	6103.2
M_T1_R2_S1_16S_S86	828.8	1.8	6280.0
M_T1_R2_S2_16S_S3	904.5	1.8	6275.8
M_T1_R2_S3_16S_S15	773.3	1.3	4368.4
M_T1_R3_S1_16S_S27	793.5	1.4	1240.0
M_T1_R3_S2_16S_S39	874.2	1.6	6191.6
M_T1_R3_S3_16S_S51	900.9	1.1	5745.3
M_T1_R4_S1_16S_S63	778.4	1.5	6970.5
M_T1_R4_S2_16S_S75	785.9	1.5	6621.1
M_T1_R4_S3_16S_S87	715.3	1.2	7770.5
M_T2_R1_S1_16S_S53	604.3	1.1	5930.5
M_T2_R1_S2_16S_S65	647.2	0.9	4781.1
M_T2_R1_S3_16S_S77	927.2	1.1	5206.3
M_T2_R2_S1_16S_S89	712.8	1.5	5012.6
M_T2_R2_S2_16S_S6	755.7	1.2	5101.1
M_T2_R2_S3_16S_S18	909.5	1.5	4696.8
M_T2_R3_S1_16S_S30	654.8	1.3	4309.5
M_T2_R3_S2_16S_S42	779.9	1.5	5623.2
M_T2_R3_S3_16S_S54	739.5	1.2	6216.8
M_T2_R4_S1_16S_S66	667.4	1.2	6646.3
M_T2_R4_S2_16S_S78	707.7	1.3	6322.1
M_T2_R4_S3_16S_S90	722.9	1.1	5265.3
M_T3_R1_S1_16S_S56	579.9	1.9	6360.0
M_T3_R1_S2_16S_S68	440.4	1.7	5458.9
M_T3_R1_S3_16S_S80	482.4	1.8	5488.4
M_T3_R2_S1_16S_S92	687.6	2.0	6018.9
M_T3_R2_S2_16S_S9	664.0	1.8	5193.7
M_T3_R2_S3_16S_S21	553.0	1.9	3837.9
M_T3_R3_S1_16S_S33	518.6	1.3	2802.1
M_T3_R3_S2_16S_S45	674.1	1.4	6052.6
M_T3_R3_S3_16S_S57	721.2	1.8	6326.3
M_T3_R4_S1_16S_S69	764.9	1.5	5867.4
M_T3_R4_S2_16S_S81	638.8	1.6	5261.1
M_T3_R4_S3_16S_S93	542.9	2.0	6772.6
M_T4_R1_S1_16S_S59	606.8	1.7	6183.2
M_T4_R1_S2_16S_S71	632.1	1.7	5442.1
M_T4_R1_S3_16S_S83	684.2	2.1	5105.3
M_T4_R2_S1_16S_S95	660.7	1.6	6010.5
M_T4_R2_S2_16S_S12	696.0	1.7	5067.4
M_T4_R2_S3_16S_S24	513.5	1.9	4444.2
M_T4_R3_S1_16S_S36	541.3	1.8	5425.3
M_T4_R3_S2_16S_S48	595.1	1.5	5437.9
M_T4_R3_S3_16S_S60	529.5	1.6	6103.2
M_T4_R4_S1_16S_S72	659.8	1.7	5631.6

M_T4_R4_S2_16S_S193	395.0	1.8	5218.9
M_T4_R4_S3_16S_S203	507.6	1.5	5113.7

Samples	POX_C	B_glucosidase	Protein
U_T1_R1_S1_16S_S1	680.0	1.1	7955.8
U_T1_R1_S2_16S_S13	717.8	1.4	8781.1
U_T1_R1_S3_16S_S25	652.2	0.9	7063.2
U_T1_R2_S1_16S_S37	574.1	0.9	7968.4
U_T1_R2_S2_16S_S49	599.3	0.7	6633.7
U_T1_R2_S3_16S_S61	614.4	0.7	6397.9
U_T1_R3_S1_16S_S73	674.9	0.8	4785.3
U_T1_R3_S2_16S_S85	606.8	0.7	6338.9
U_T1_R3_S3_16S_S2	624.5	0.7	7934.7
U_T1_R4_S1_16S_S14	677.5	1.0	6490.5
U_T1_R4_S2_16S_S26	662.3	0.9	8953.7
U_T1_R4_S3_16S_S38	677.5	1.4	9016.8
U_T2_R1_S1_16S_S4	778.4	0.8	6541.1
U_T2_R1_S2_16S_S16	869.2	0.7	7277.9
U_T2_R1_S3_16S_S28	894.4	0.9	4414.7
U_T2_R2_S1_16S_S40	639.6	0.6	4924.2
U_T2_R2_S2_16S_S52	859.1	0.7	4317.9
U_T2_R2_S3_16S_S64	738.0	0.9	5576.8
U_T2_R3_S1_16S_S76	876.7	1.0	5341.1
U_T2_R3_S2_16S_S88	846.5	1.0	2477.9
U_T2_R3_S3_16S_S5	611.9	1.1	7189.5
U_T2_R4_S1_16S_S17	859.1	1.2	7206.3
U_T2_R4_S2_16S_S29	637.1	1.2	6187.4
U_T2_R4_S3_16S_S41	711.3	1.0	6073.7
U_T3_R1_S1_16S_S7	504.3	0.9	7088.4
U_T3_R1_S2_16S_S19	407.6	0.8	5972.6
U_T3_R1_S3_16S_S31	273.9	0.7	1021.1
U_T3_R2_S1_16S_S43	571.5	0.8	6309.5
U_T3_R2_S2_16S_S55	551.4	0.8	6545.3
U_T3_R2_S3_16S_S67	588.3	1.0	4848.4
U_T3_R3_S1_16S_S79	377.3	1.0	3004.2
U_T3_R3_S2_16S_S91	289.1	0.7	4524.2
U_T3_R3_S3_16S_S8	707.7	1.2	5383.2
U_T3_R4_S1_16S_S20	392.5	1.1	6212.6
U_T3_R4_S2_16S_S32	536.2	0.7	5774.7
U_T3_R4_S3_16S_S44	677.5	0.7	8317.9
U_T4_R1_S1_16S_S10	585.0	1.2	6915.8
U_T4_R1_S2_16S_S22	554.7	1.3	5589.5
U_T4_R1_S3_16S_S34	894.4	1.0	5972.6
U_T4_R2_S1_16S_S46	692.6	1.5	6747.4
U_T4_R2_S2_16S_S58	751.5	1.3	6448.4
U_T4_R2_S3_16S_S70	568.2	1.4	5075.8
U_T4_R3_S1_16S_S82	473.2	1.0	3416.8
U_T4_R3_S2_16S_S94	210.9	1.0	5480.0
U_T4_R3_S3_16S_S11	321.8	1.1	5922.1
U_T4_R4_S1_16S_S23	632.1	1.1	2587.4

U_T4_R4_S2_16S_S35	674.1	1.2	5383.2
U_T4_R4_S3_16S_S47	381.5	1.1	7816.8
